# Study of the inhibition effects on glutathione peroxidase immobilized on MNPs using a stopped-flow microfluidic system

**DOI:** 10.1007/s00216-023-04521-0

**Published:** 2023-01-18

**Authors:** Vanesa Román-Pizarro, Alba María Carrión-Escudero, Ángela Écija-Arenas, Juan Manuel Fernández-Romero

**Affiliations:** Departamento de Química Analítica, Instituto Universitario de Investigación en Química Fina Y Nanoquímica (IUNAN), Universidad de Córdoba, Edificio Anexo “Marie Curie”, Campus de Rabanales, 14071 Córdoba, Spain

**Keywords:** Glutathione peroxidase, Stopped-flow microfluidic, Enzyme-magnetic nanoparticles, Bifurcated fiber optic fluorimetry, Competitive redox reaction, Metallic ions and hydroperoxide as inhibitors

## Abstract

A stopped-flow microfluidic system to monitor glutathione peroxidase (GPx) activity and evaluate potential inhibitors of the enzyme has been developed based on the integration of the microfluidic chip in the reaction/detection zone. This integration supposes the physical alignment at the optimal location of the microfluidic channel, both the magnetically retained enzyme microreactor (MREµR) and the remote luminescence detection using a focused bifurcated fiber optic bundle (BFOB) connected to a conventional spectrofluorometer detector. The method is based on the coupling of two competitive oxidative chemical reactions, in which glutathione (GSH) and homovanillic acid (HVA) competed for their interaction with hydrogen peroxide in the presence of the magnetically retained GPx-MNPs. The biocatalytic reaction was followed by monitoring the fluorescence of the biphenyl-HVA dimer formed. The dynamic range of the calibration graph was 0.45–10 µmol L^−1^, expressed as GSH concentration with a detection limit of 0.1 µmol L^−1^ (*r*^2^ = 0.9954, *n* = 10, *r* = 3). The precision expressed as the relative standard deviation (RSD%) was between 0.5 and 3.9%. The stopped-flow microfluidic system showed a sampling frequency of 25 h^−1^. The method was applied to the study of GPx inhibition provided by three inhibitory compounds, two metallic ions Hg(II) and Cu(II) and t-butyl hydroperoxide, and their presence in liquid samples, as water, milk, and edible oil. Recovery values between 88.7 and 99.4% were achieved in all instances.

**Graphical Abstract:**

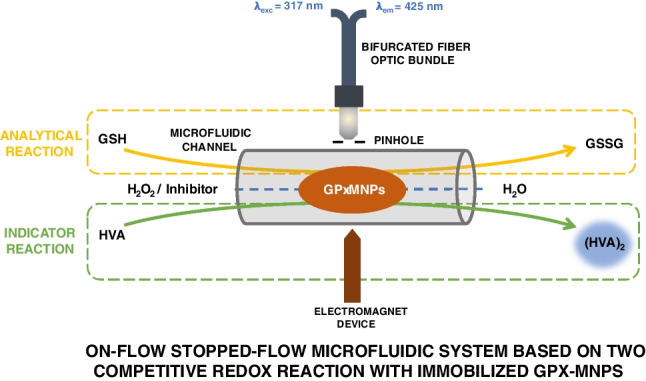

## Introduction

Several analytical methods have been based on kinetic systems applying enzymes in solution and immobilized in areas such as clinical biochemistry [1–5]. Some of the characteristics inherent to developing analytical determinations based on the use of enzymes are sensitivity and selectivity. However, one of the most significant limitations is the cost of its use in the solution. Therefore, enzyme immobilization is supposed some advantages, such as reusability, resistance to moderate changes in pH, temperature, or ionic strength of the medium, and the ability to maintain the enzymatic activity stable over time [6–8]. The immobilization of enzymes on inert supports and their localization in small reactors in the reaction/detection zone is the most common way to incorporate enzymatic biocatalysts in continuous automatic analysis systems. These have been arranged in the form of reactors with immobilized enzymes using controlled pore glass (IMER) or, more recently, by magnetic retention technology in the form of a magnetically retained enzyme reactor (MRER). In case the flow systems consisted of microfluidic, the dimensions of the reactor decrease, considering the magnetically retained enzyme microreactor (MREµR). To obtain this type, enzymes were immobilized on magnetic nanoparticles (MNPs) [9, 10].

The use of enzymes with peroxidase activity has been widely used in the field of analytical chemistry when they are incorporated as immobilized biocatalysts [11]. Usually, these enzymes with peroxidase activity catalyze the oxidation of organic and inorganic compounds in the presence of hydrogen peroxide (H_2_O_2_), which acts as an oxidant. The enzyme glutathione peroxidase (glutathione:hydrogen-peroxide oxidoreductase; EC1.11.1.9, GPx) is an enzyme belonging to the family of seleno-proteins that catalyzes the reduction of peroxides using glutathione (GSH) as a reductor [12]. The GPx enzyme is one of the most important within this group of enzymes due to its role as an antioxidant in defense as a protector against reactive oxygen species (ROS) [13, 14].

For the determination of glutathione in biological fluids, different photometric-enzymatic methods have been proposed using the immobilized GPx enzyme [15]. On the other hand, several methods have been developed that involve the characterization of activators and inhibitors of the GPx enzyme, and these methods can be enzymatic with fluorometric detection or photometric [16–19]. Many of them involve the determination of GPx inhibitors by vitamins, metals, cysteine, selenite, and hydroperoxides [20–26]. The most used method for determining inhibitors of the GPx enzyme is based on the photometric monitoring of the oxidation of GSH to GSSG [22].

In this work, an auxiliary reaction was used to carry out the fluorimetric monitoring of the enzymatic reaction. The signal obtained with this reaction indicates the competitive reaction with the analytical reaction catalyzed by the enzyme glutathione peroxidase. The oxidation of homovanillic acid (HVA) with H_2_O_2_ has been used as a secondary reaction [27, 28].

Finally, the development of this enzymatic method at microfluidic size has been performed by using an on-flow integrated stopped-flow mode, given its usefulness in the development of kinetic-enzymatic methodologies in clinical biochemistry, especially those in which biocatalysts have been immobilized. Microfluidic devices are supposed an alternative to reduce enzyme consumption and ease immobilization [29]. The integrated microfluidic system is based on the on-flow stopped-flow kinetic model, in which the GPx-MNPs complex has been magnetically retained in the reaction/detection zone, where the biocatalytic reaction that competes with the oxidation reaction of HVA took place.

This work demonstrates a method based on a stopped-flow microfluidic system using GPx immobilized in the reaction/detection zone. The developed microfluidic system has been applied to the determination of glutathione as a substrate for the enzyme glutathione peroxidase and the study of potential glutathione reductase inhibitors in the environment and food samples. In this double system of redox reactions, the substrates of both GSH and HVA reactions compete for their interaction with H_2_O_2_, and the luminescent signal could be related to the presence of potential inhibitor compounds, such as metals Hg(II) and Cu(II) and the t-butyl hydroperoxide. In the case of the inhibition due to the presence of the metals Cu(II) and Hg(II), the development of the biocatalytic reaction at basic pH conditions induced the formation of metal-enzyme complexes preventing the formation of the enzyme–substrate complex, promoting a competitive inhibition [24, 30]. For t-butyl hydroperoxide, it could be a non-competitive inhibitor since it prevented the GSH oxidation by H_2_O_2_ at the studied concentration levels, even if it is a substrate of the enzymatic reaction [30, 31].

## Materials and methods

### Materials

All chemicals used were of analytical grade. Hydrogen peroxide at 30% w/v (H_2_O_2_), and iron(III) chloride (FeCl_3_) were supplied by Panreac (PANREAC Química S.L.U., Barcelona, Spain, https://www.itwreagents.com/iberia/es/home). Homovanillic acid (HVA), iron(II) chloride (FeCl_2_), N-(3-dimethyl aminopropyl)-N′-ethyl carbodiimide (EDC), glutathione (GSH), tert-butyl hydroperoxide, mercury(II) chloride (HgCl_2_), and copper(II) sulfate (CuSO_4_) were supplied by Sigma-Aldrich (Merck Life Science S.L.U., Madrid, Spain, http://www.sigmaaldrich.com/ES/es). Sodium hydroxide (NaOH), hydrochloric acid (HCl), disodium phosphate, and Tris–HCl were provided by Merck (Merck Life Science S.L.U., Madrid, Spain, http://www.sigmaaldrich.com/ES/es). The enzyme glutathione peroxidase (GPx) (EC.1.11.1.9) (glutathione peroxidase from bovine blood erythrocyte samples), with an activity of approximately 300 U mg^−1^ protein (expressed in DEA units), was also supplied by Sigma-Aldrich.

### Apparatus and instruments

All fluorescence signals were acquired using an FL-3000/FM4-3000 bifurcated fiber optic bundle (BFOB) assembled to a Horiba Scientific Fluoromax-4P spectrofluorometer (Horiba Scientific, France, www.horiba.com/scientific/). The information provided by the instrument was processed by FluorEssence Software (HoribaScientific) and OriginPro 2018 64-bit Software (OriginLab Co. 2018, Northampton, MA). The microfluidic system was adapted to the spectrofluorimeter and aligned to the fiber optic focal point using an x–y-z displacement device (Oriel Instruments, USA, www.newport.com/oriel/). The flow was driven using a Gilson Minipuls-2 peristaltic pump (Gilson Inc., Middelton, UK, www.gilson.com) and a KDS220 syringe pump (KD Scientific Inc., MA, USA, www.kdscientific.com).

As the microfluidic device, a borofloat glass microreactor (H300.015.2) with 15 × 45 mm dimensions and a 2.75 µL internal volume was inserted into a chip holder (FC-PRO.CH4515) (Micronit, Netherlands, www.micronit.com). A lab-built electromagnet facilitated the magnetic retention on the focal point. A Raspberry Pi 3 Model B (Raspberry Pi Foundation, United Kingdom, www.desingspark.com/raspberrypi) as an embedded system has been used to control the electromagnet device using Phyton 3 commands. Two Cheminert VA-CN2 injection valves (Valco, Teknokroma, Barcelona, Spain, www.teknokroma.es) were used to inject both the GPx-MNPs complex and the other reactant solutions. Different polytetrafluoroethylene (PTFE) connections and tubes of 1/16″ outer diameter, 250 μm inner diameter, perfluoro-elastomer splints (FFKM), and adequate PEEK connectors have been used.

A conventional oven, an ultrasound bath, and an MPW-350R centrifuge (MPW Med. Instrument, Warsaw, Poland, www.mpw.pl) with a cooling chamber rotating and an angle rotor HSL-11199 (45°, 12 × 12 × 1.5 mL, max. speed = 18,000 rpm, 24,088 × *g* RCF and *r*_min_/*r*_max_ = 3.5/6.25) were used for the GPx-MNPs synthesis.

### Selection of the enzymatic system

**Fig. 1:**
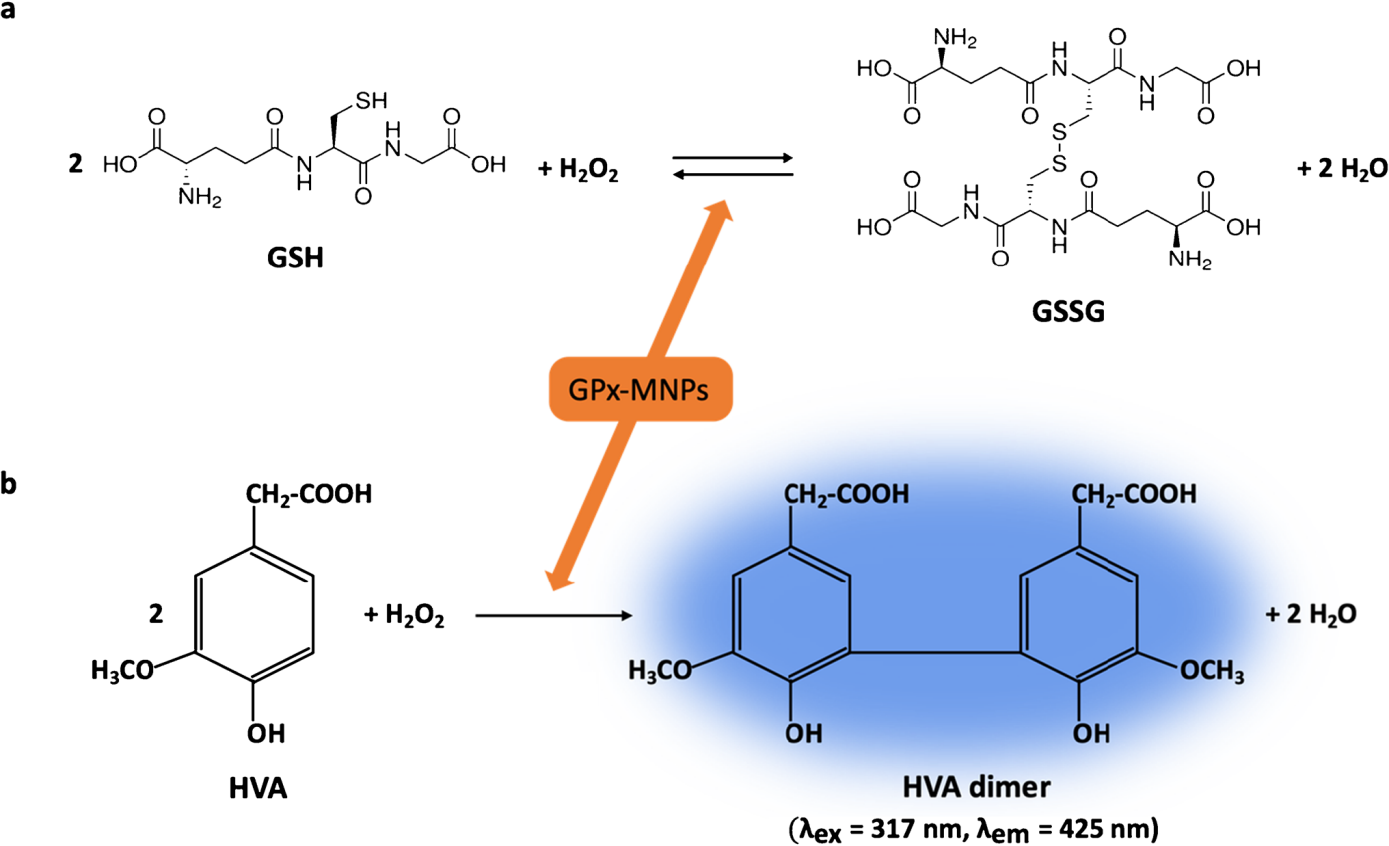
Reactions involved in the proposed method. **a** Analytical reaction in which glutathione (GSH) oxidation was catalyzed by the glutathione peroxidase (GPx) enzyme to form GSH disulfide (GSSG). **b** Indicator reaction in which homovanillic acid (HVA) dimer formation occurred for the luminescent monitoring of the enzymatic reaction

The chemical reaction selected for this study involved the reduction of H_2_O_2_ to water using the GSH as a reducing compound, which, in turn, competes with the HVA. The analytical reaction studied in this method was the GSH oxidation, but for that, an indicator reaction in which the dimerization of HVA occurred was needed. HVA reaction was used to monitor the reaction by fluorimetry, as shown in Fig. 1. HVA reacted with H_2_O_2_, giving rise to a fluorescent dimer, with excitation and emission wavelengths of 315 and 425 nm, respectively, at a pH higher than 8 [28]. The decrease in the fluorescence signal can be related to the concentration of peroxide compounds present in the sample.

### Synthesis of GPx-MNPs complex

To perform the synthesis of the GPx-MNPs complex, a previous step for the MNPs synthesis was needed. This synthesis was carried out using the previously described co-precipitation method [32]. Before their use for enzyme immobilization, the solvent was evaporated until completely dried in an oven at 90 °C until the weight remained constant in five successive weightings. The immobilization of the GPx enzyme was carried out by a covalent binding using the cross-linking carbodiimide reaction, which modified the immobilization of other enzymes [8, 33, 34]. Initially, 0.0498 g of dried MNPs were added to 1 mL of phosphate buffer (50 mmol L^−1^, pH 7.4), and the solution was sonicated for 15 min after adding 0.5 mL of a 0.02 g mL^−1^ EDC solution dissolved in the phosphate buffer mentioned. After this, 2 mL of a 100 U mL^−1^ enzyme solution in Tris–HCl buffer (50 mmol L^−1^, pH 9) is added and introduced again in the ultrasonic bath for 30 min at 4 °C. The suspension with the enzyme immobilized was centrifuged at 3000 rpm for 20 min. Subsequently, three washes were performed: two with a 50 mmol L^−1^ phosphate buffer adjusted to pH 8, and one with a 100 mmol L^−1^ Tris–HCl buffer to which 100 mmol L^.1^ NaCl was added and was adjusted to pH 8. Supernatants obtained were stored in the fridge at 4 °C until their use in the measurement of the immobilization effectiveness.

The characterization of the GPx-MNPs complex has been avoided because MNPs and the complex with enzymes have been previously described [32, 34]. After the immobilization of the enzyme, “batch” tests were performed to evaluate the efficiency of the GPx immobilization process on MNPs. These studies consisted in the measure of the fluorescence intensity of the reaction that occurred between HVA (0.1 mmol L^−1^) and H_2_O_2_ (0.1 mmol L^−1^) in presence of GSH (2.5 µmol L^−1^) and different GPx enzyme activities (0–1 U mL^−1^). All solutions were prepared in the 50 mmol L^−1^ Tris–HCl buffer adjusted at pH 9. In all the trials, the signal reduction at the excitation and emission wavelengths of the HVA dimer was monitored. The GPx immobilization efficiency using the fluorimetric method described above let results in an immobilization yield of 92.1 ± 0.5%. The behavior of the immobilized enzyme has been compared to that of the enzyme in solution, with the same activity enzyme immobilized and in solution (0.5 U mL^−1^). Similar enzyme activity was obtained in both cases, assuming no activity loss by immobilizing the enzyme. The stability of the immobilized enzyme has also been evaluated by monitoring the signals of decreased fluorescence over time. All the tests were conducted under experimental conditions with different concentrations of HVA (0.1–10 μmol L^−1^). The stability of the immobilized enzyme was maintained constant for at least 2 months when it was immobilized (activity losses lower than 2%).

### Microfluidic system configuration

**Fig. 2:**
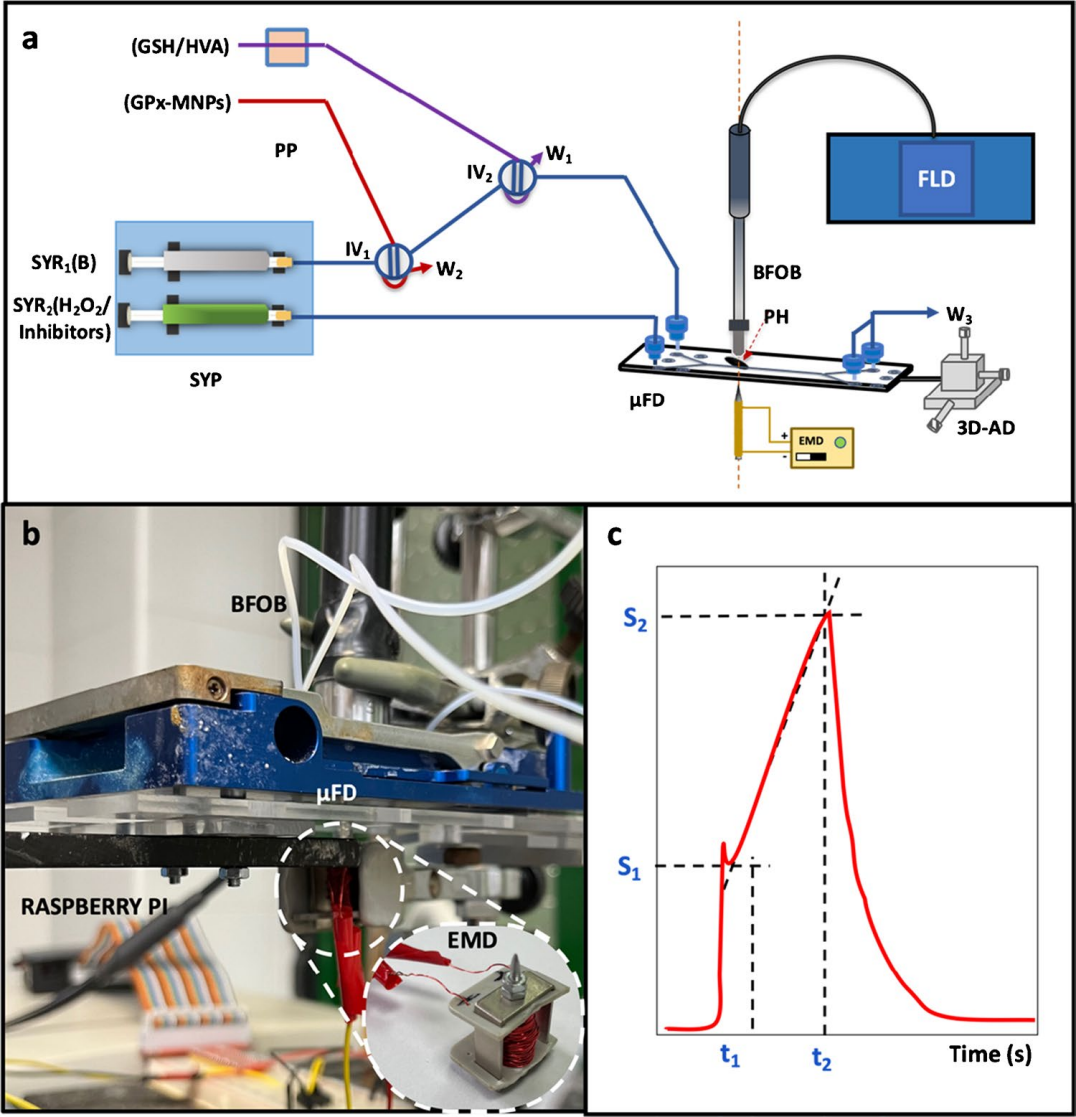
**a** Diagram of the integrated on-flow stopped-flow microfluidic system used. **b** Detail of the integration of the electromagnet device and the bifurcated fiber optic bundle with the microfluidic device in the reaction/detection zone. **c** Instrumental signals acquired with the stopped-flow microfluidic system. PP denotes peristaltic pump; SYP, syringe pump; SYR_1_, syringe to introduce buffer solution; SYR_2_, syringe to introduce H_2_O_2_ and inhibitor compounds; IV_1_, injection valve for the GPx-MNPs complex solution; IV_2_, injection valve for the mixture GSH/HVA solution; µFD, microfluidic device; BFOB, bifurcated fiber optic bundle; PH, pinhole; EMD, electromagnet device; 3D-AD, x–y-z alignment device; FLD, fluorimetric detector; *W*_1_, *W*_2_, and *W*_3_ waste; *S*_1_ and *S*_2_, signals after the injection and at the end of the kinetic curve; *t*_1_ and *t*_2_, injection and stopped time

The microfluidic size of the system with an on-flow stopped-flow mode was chosen to develop the competitive kinetic reactions. For this purpose, the GPx enzyme was incorporated and retained in the reaction/detection zone of the system as GPx-MNPs complex to form the MREµR. This device acted as a catalytic enzyme reactor but also provides a rapid mixture of the reactants avoiding laminar diffusion. Figure 2a depicts the on-flow stopped-flow microfluidic configuration. Figure 2b shows an image in detail of the coupling of the lab-built electromagnet device used to retain the immobilized enzyme and form the MREµR, as well as the coupling of a BFOB in the reaction/detection zone to monitor the reaction. The control of the electromagnet device has been carried out using the embedded device Raspberry Pi 3 Model 3 with Phyton commands. The BFOB has been aligned to the reaction/detection zone of the microfluidic device using an x–y-z alignment device. As can be seen, the use of a microfluidic device incorporating two injection valves has been chosen to control the regeneration of the MREµR and introduce simultaneously the two reducing substrates of both analytical (GSH) and indicator (HVA) reactions. H_2_O_2_ was introduced continuously as the oxidant substrate to both reactions towards the reaction/detection zone. When a GPx inhibitor is required, this was introduced in the same solution with the oxidant substrate.

One first stage was required to introduce a volume of 50 µL solution containing the GPx-MNPs complex through the injection valve (IV_1_) to form the MREµR, leaving the enzyme magnetically retained in the optical pathway. This step is required once for all measurements since the enzyme was reused. In the second stage, the injection of the GSH/HVA substrate mixture through injection valve IV_2_ into the reaction/detection zone occurred. At an exact previous injection time of 10 s (*t*_1_), since the substrates were injected, the flow was stopped, and the kinetic tracking of the enzymatic reaction took place during the stopped time of 120 s (*t*_2_), in which a proportional peak corresponding to the development of both competing reactions (enzyme-catalyzed and indicator) was formed. The hydrogen peroxide solution and the inhibitors passed continuously through the system.

The reaction of the HVA with the H_2_O_2_ catalyzed by GPx caused an increase in the fluorescence due to the HVA dimer, which is monitored in the spectrofluorimeter at the excitation and emission wavelengths of 317 and 425 nm, respectively, using an ex/em slits rate of 5/5 with PMT gain of 950 V. Two types of signals were processed: (a) net signals obtained as the difference between the absence (*I*_0_) and the presence (*I*) of GSH, being each one the difference between the end (*S*_2_) and the beginning (*S*_1_) of the kinetic curve; and (b) kinetic signals of reaction rates. The signals acquired, as shown in Fig. 2b, showed the typical stopped-flow signal, whose intensity decayed proportionally to the GSH concentration introduced since HVA and GSH competed to reduce H_2_O_2_. When the study of inhibitors was carried out, their presence induced a signal increase due to inhibiting the glutathione reduction. In this case, the type of enzymatic inhibition was studied thanks to the double reciprocal representation established by Lineweaver–Burk, and the concentration of those inhibitors was determined in different samples.

### Sample analysis

The method was applied to determine three different inhibitors of the GPx enzyme, such as Hg(II), Cu(II), and t-butyl hydroperoxide. These compounds were analyzed in samples of different natures, such as tap water, well water, whole milk, and edible oil, the last two obtained from supermarkets. Sample pretreatment was carried out as follows [35]. The milk samples were pretreated by mixing 20 mL of each sample with 20 mL of 20% trichloroacetic acid solution, stirred for 40 min, and subjected to centrifugation at 4 °C for 15 min at 11,830 × g. Obtained supernatants were collected and filtered through a 0.2 µm membrane, followed by dilution fivefold with Tris–HCl buffer (50 mmol L^−1^, pH 9). The oil samples were weighted (15 g) and homogenized in 75 mL n-hexane, the mixture was evaporated at 60 °C using a rotary evaporator, and the dried residue was dissolved in 7.5 mL of 5% chloroform. In both cases, the resulting extract solutions were adequately diluted with Tris–HCl buffer for their introduction into the flow system. Two dilution factors were used, 1:100 for the oil samples and 1:10 for the milk samples. The water samples did not need pretreatment, and no dilution was performed in them.

## Results and discussion

### Study of the variables involved in the developed system

Table 1 summarizes the instrumental, physical, hydrodynamic, and chemical variables studied, using the univariate method, and performing at least three repetitions for each assay, the ranges assayed, and the values chosen. All measurements were studied using two GSH concentrations, 1 and 5 µmol L^−1^, and 0.5 U mL^−1^ activity of the immobilized GPx enzyme. The net signal of the fluorescence intensity corresponding to the *I*_0_ − *I* differences was selected as the measurement parameter, the intervals tested, and the optimal values, or those adopted due to compromise with the enzymatic and instrumental system.

**Table 1:** Optimization of variables

Type of variable	Variable	Range studied	Optimal value
Instrumental	*λ*_ex_, nm	200–800	317
	*λ*_em_, nm	200–800	425
	Excitation slits, nm	1–10	5
	Emission slits, nm	1–10	5
	PMT power, V	600–950	950
	Focused length, mm	2–20	10
	Pinhole diameter, mm	0.1–3	1.5
	Electromagnet device power, V	–	6
	Electromagnet device tip length, mm	1–10	5
Physical	Temperature, °C	25–60	30
Hydrodynamic	Flow rate, µL min^−1^	10–100	40
	Injection volume IV_1_, µL	10–50	50
	Injection volume IV_2_, µL	1–5	5
	Previous time, s	5–20	10
	Stopped time, s	50–200	120
Chemical	[Tris–HCl], mmol L^−1^	10–100	50
	pH	6–9	9
	GPx-MPNs dilution	1/2–1/10	1/5
	[H_2_O_2_], µmol L^−1^	0.1–10	10
	[HVA], µmol L^−1^	1–10	2.5

**Fig. 3:**
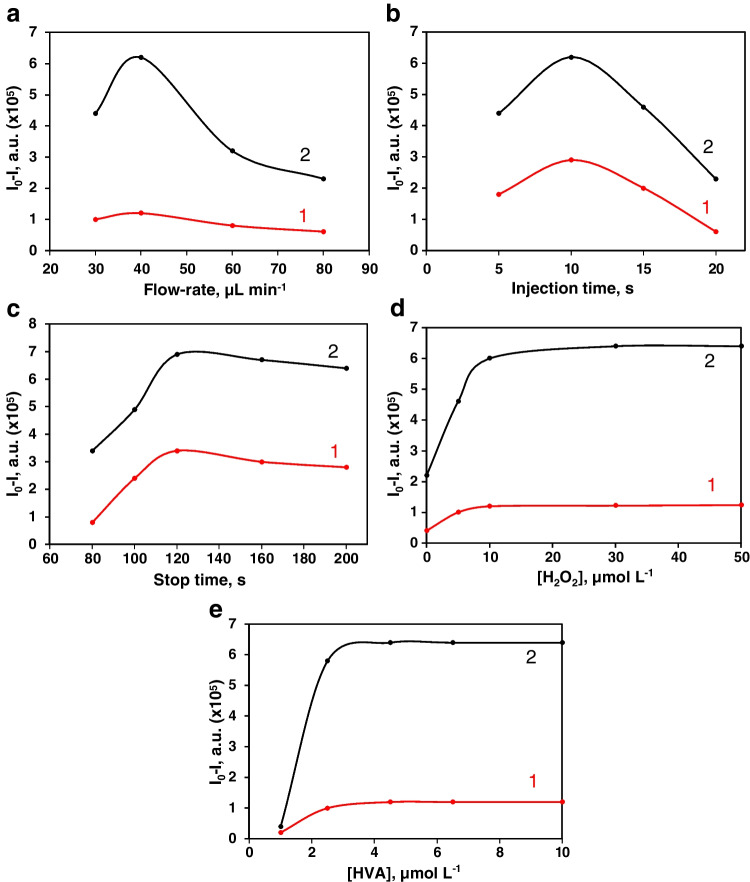
Influence of some experimental variables of the stopped-flow microfluidic system: **a** flow rate, **b** previous time, **c** stopped time, **d** [H_2_O_2_], and **e** [HVA]. The net signal (*I*_0_ − *I*) obtained is shown for two concentrations of GSH, (1) 1 and (2) 5 µmol L^−1^

The study of the instrumental variables was carried out with the spectroscopic study of the HVA indicator reaction. The focus distance of the fiber optic bundle and the pinhole adapted to focus excitation beam at the microfluidic channel were set up to obtain the best signal from the MREµR. The power and the length of the electromagnet device inserted to retain the GPx-MNPs complex were studied to obtain the smallest reactor with the maximum enzymatic capacity. The optimal temperature influence was achieved at 30 °C, and values above this temperature caused denaturation of the enzyme. Concerning the hydrodynamic variables, the flow had a significant influence to provide the rapid displacement of the reagents to the reaction/detection zone. Figure 3a shows the flow rate studied, obtaining the best results with a 40 µL min^−1^ flow rate.

As already mentioned, the microfluidic configuration included two injection valves. IV_1_ incorporated the GPx-MNPs complex to form the MREµR, choosing an injection volume of 50 µL to retain as much of the immobilized enzyme as possible and to obtain a short segment of MREµR to avoid clogging problems. A 1:5 diluted suspension in phosphate buffer (50 mmol L^−1^, pH 7.4) was incorporated. Concerning IV_2_, the reactants involved in enzymatic and indicator reactions were introduced, with a volume of 5 µL to obtain a narrow peak. Figure 3b and c show the influence of the previous time and the stopped time, observing the best signals at the optimal values of 10 and 120 s, respectively.

Furthermore, to study the chemical variables, a kinetic study of the two oxidation reactions with the GSH and HVA substrates in presence of H_2_O_2_ was performed. The best results were obtained using Tris–HCl buffer 50 mmol L^−1^ adjusted to pH 9. One of the fundamental steps was the conversion of GSH to GSSG, since the H_2_O_2_ was consumed, and the rest reacted in the indicator reaction with HVA. For this, enough H_2_O_2_ must be added so that once it reacts with GSH, there was enough left to give the indicator reaction. As shown in Fig. 3d, for levels of H_2_O_2_ concentration higher than 10 µmol L^−1^, the net signal was not modified. The HVA concentration should be studied to obtain an extended range of linearity for the method without the saturation of the indicator reaction. Figure 3e depicts the best signal acquired with 2.5 µmol L^−1^ for HVA.

### GPx enzyme inhibition study in the microfluidic system

The study of the potential inhibition effect of two metals (Cu(II) and Hg(II)) and t-butyl hydroperoxide was carried out to demonstrate the usefulness of the stopped-flow microfluidic system. In all cases, the representation of the initial rate (*v*_i_) using the double reciprocal method of Lineweaver–Burk was established. The determination of the inhibition constant of each specie was carried out at three concentrations of each inhibitor compound (1, 5, and 10 µmol L^−1^), and this study allowed knowing the inhibition model of each tested compound. The results obtained for each inhibitor compound are listed in Table 2. As can be deduced, the metals Hg(II) and Cu(II) behave as competitive inhibitors because the *v*_MAX_ values remained constant (estimated value of 715.7 ± 3.6 µmol L^−1^ s^−1^) and *K*_M_ increased depending on the metal concentration. In the case of t-butyl hydroperoxide, the values of *K*_M_ (estimated value of 593.79 ± 7.98 µmol L^−1^) remained practically constant, and there was a slight decrease in *v*_MAX_ at increasing inhibitor concentration, confirming the non-competitive inhibition. The inhibition observed showed similar results to those obtained in the literature [24, 30, 31], meaning that the immobilization of the enzyme in MNPs does not affect the inhibition response of the enzyme.

**Table 2:** Kinetic-enzymatic parameters that define the GPx enzyme inhibition

Inhibitor type	[Inhibitor], µmol L^−1^	Lineweaver–Burk equation			Kinetic parameters		*K*_I_, µmol L^−1^ s^−1^
		Slope	Intercept	*r*^2^	*K*_M_, µmol L^−1^	*v*_MAX_, µmol L^−1^ s^−1^	
Hg(II)	1	0.01	0.0015	0.998	46.98	712.71	749.18
	5	0.064	0.0014	0.997	71.42	714.32	1023.30
	10	0.128	0.0013	0.966	91.66	720.09	1323.80
Cu(II)	1	1.59	0.002	0.991	649.71	153.98	1129.65
	5	5.35	0.003	0.992	744.32	141.49	4234.98
	10	1.33	0.0007	0.994	2530.09	149.66	23,190.9
t-butyl hydroperoxide	1	2.27	0.06	0.998	580.09	158.98	117.09
	5	7.37	0.13	0.987	599.65	138.09	293.09
	10	15.76	0.04	0.965	601.65	121.98	2803.45

### Features of the method

Once the inhibition effect has been demonstrated, the method was applied to determine these inhibition compounds. For that, some calibration graphs were found using the optimal values obtained in the previous section and using net signals. A calibration graph was obtained to determine the substrate, and other ones were acquired to determine Cu(II), Hg(II), and t-butyl hydroperoxide. Table 3 shows the equation parameters of the calibration graphs, the concentration ranges studied, the detection limit calculated according to the IUPAC recommendations, and the precision expressed in terms of relative standard deviation percentage (RSD%).

**Table 3:** Features of the method

	Equation parameters^a^			LOD, µmol L^−1^	Linear range, µmol L^−1^	RSD, %^b^	
	Slope	Intercept	*r*^2^			Max. error	Min. error
Glutathione	2.9·10^4^ (± 2·10^2^)	4.5·10^5^ (± 1.6·10^4^)	0.9954	0.1	0.45–10	3.9	0.6
Hg (II)	3.1·10^3^ (± 54.5)	3.1·10^3^ (± 3.2·10^2^)	0.9969	0.031	0.1–10	5.4	1.1
Cu (II)	1.7·10^4^ (± 4.3·10^2^)	1.9·10^4^ (± 2.7·10^2^)	0.9993	0.049	0.2–10	4.3	0.9
t-butyl hydroperoxide	3.1·10^4^ (± 3.1·10^2^)	3.6·10^4^ (± 2.1·10^2^)	0.9902	0.019	0.06–10	4.8	0.8

^a^*y* = (*I*_0_ − *I*) and *x* = [GSH], µmol L^−1^

^b^RSD% values achieved at the maximum and minimum error zones (0.5 µmol L^−1^ and 5 µmol L^−1^, respectively)

### Application of the method

The method was applied to determine some inhibitors of the GPx enzyme in environmental and food samples, such as tap water, well water, milk, and edible oil, to establish the applicability of the on-flow stopped-flow microfluidic system developed using the procedure described above. The pretreated and diluted samples were first analyzed to verify the presence of the inhibition compounds. However, some of the studied compounds were not contained in the samples. Therefore, a recovery study was carried out by adding two different amounts of each compound to each sample to achieve final analyte concentrations of 1 and 5 µmol L^−1^. Table 4 shows the recovery values obtained, ranging between 88.7 and 99.4%. The results confirmed that the developed method was highly reliable and applicable to Hg(II), Cu(II), and t-butyl hydroperoxide detection in real samples.

**Table 4:** Application of the method

Sample	Studied compound	Found^a^	Recovery^a,b^	
			1st add	2nd add
Tap water	Hg(II)	n.d.^c^	98.3	94.8
	Cu(II)	n.d.^c^	95.2	90.9
Well water	Hg(II)	n.d.^c^	94.6	93.7
	Cu(II)	n.d.^c^	99.4	96.2
Whole milk	t-butyl hydroperoxide	0.9 ± 0.02	95.5	92.8
Olive oil	t-butyl hydroperoxide	8.6 ± 0.05	93.4	89.9
Sunflower oil	t-butyl hydroperoxide	28.9 ± 0.09	94.2	88.7

^a^Mean value of three experiments

^b^1st adds, 1 µmol L^−1^ and 2nd add, 5 µmol L^−1^

^c^Non-detected

## Conclusions

A stopped-flow microfluidic device has been developed successfully using a magnetically retained enzymatic microreactor to study kinetic-enzymatic parameters and the inhibitory properties of some compounds in the enzyme. For that, glutathione peroxidase (GPx) has been immobilized on magnetic nanoparticles (MNPs) to form the GPx-MNPs complex to be retained in the reaction/detection zone of the microfluidic system. A chemical system has been proposed for monitoring the GPx enzymatic activity in the absence and presence of inhibitors, based on the combination of two oxidation reactions in which the enzyme substrate, glutathione, and homovanillic acid competed for their interaction with H_2_O_2_. The product of the homovanillic acid reaction was a fluorescent compound that allowed the quantification of glutathione or inhibitor compounds. Finally, developing a microfluidic system based on the stopped-flow mode allowing enzyme retention is a useful tool to characterize enzymatic systems and study the behavior of enzymatic inhibitors with low enzyme consumption.
